# Reactive oxygen species regulate leaf pulvinus abscission zone cell separation in response to water-deficit stress in cassava

**DOI:** 10.1038/srep21542

**Published:** 2016-02-22

**Authors:** Wenbin Liao, Gan Wang, Yayun Li, Bin Wang, Peng Zhang, Ming Peng

**Affiliations:** 1 Institute of Tropical Bioscience and Biotechnology, Chinese Academy of Tropical Agricultural Sciences, Haikou 571101, China; 2 National Key Laboratory of Plant Molecular Genetics, CAS Center for Excellence in Molecular Plant Sciences, Institute of Plant Physiology and Ecology, Shanghai Institutes for Biological Sciences, Chinese Academy of Science, Shanghai 200032, China

## Abstract

Cassava (*Manihot esculenta* Crantz) plant resists water-deficit stress by shedding leaves leading to adaptive water-deficit condition. Transcriptomic, physiological, cellular, molecular, metabolic, and transgenic methods were used to study the mechanism of cassava abscission zone (AZ) cell separation under water-deficit stress. Microscopic observation indicated that AZ cell separation initiated at the later stages during water-deficit stress. Transcriptome profiling of AZ suggested that differential expression genes of AZ under stress mainly participate in reactive oxygen species (ROS) pathway. The key genes involved in hydrogen peroxide biosynthesis and metabolism showed significantly higher expression levels in AZ than non-separating tissues adjacent to the AZ under stress. Significantly higher levels of hydrogen peroxide correlated with hydrogen peroxide biosynthesis related genes and AZ cell separation was detected by microscopic observation, colorimetric detection and GC-MS analyses under stress. Co-overexpression of the ROS-scavenging proteins SOD and CAT1 in cassava decreased the levels of hydrogen peroxide in AZ under water-deficit stress. The cell separation of the pulvinus AZ also delayed in co-overexpression of the ROS-scavenging proteins SOD and CAT1 plants both *in vitro* and at the plant level. Together, the results indicated that ROS play an important regulatory role in the process of cassava leaf abscission under water-deficit stress.

The ability of a plant to shed unnecessary leaves is essential to maximize viability, whether the leaves are abscised at the end of a growing season or prematurely as a means of plant defense^1^,^2^, which reduces yield and quality of crop plants^3^. During the process of leaf abscission, cell separation occurs at the site of several layers of densely cytoplasmic cells, called the abscission zone (AZ)^1^. Understanding the mechanism of leaf abscission and avoiding premature leaf abscission can buoy the yield and quality of crop plants.

Cell separation is a critical activity during organ abscission^4^. Cell separation will be triggered by environmental or hormonal signals or by a switch when a cell has reached a specific developmental stage^4^. The abscission zone in many species is morphologically distinguishable before evocation of the cell separation process, cell separation occurs at a predetermined region where small and dense cells form layers, and the cells will be swollen when abscission is initiated^4^,^5^. In addition, cell transdifferentiation event also was discovered in bean, the mature cortical cells will convert to functional abscission cells by exogenous ethylene and auxin treatment^5^.

The study of plant organ abscission mainly focuses on flower abscission, fruit abscission and leaf abscission^6^. The confirmed mechanism of plant organ abscission occurs as the result of altered hormone signaling, mainly ethylene and auxin^7^. Global analyses have identified the dynamic changes during flower abscission and fruit abscission^8^. Microarray analysis of transcriptome changes in tomato flowers (*Solanum lycopersicum* ‘Shiran 1335’) with or without preexposure to 1-methylcyclopropene or application of indole-3-acetic acid after flower removal indicated that acquisition of ethylene sensitivity in the AZ is associated with altered expression of auxin-regulated genes resulting from auxin depletion. This suggested that ethylene and auxin homeostasis regulates flower abscission after flower removal^6^. Transcriptomic profiles of persisting and abscising fruitlets were performed to study fruitlet abscission with exogenous benzyladenine (BA) treatment. The results indicated that fruitlet abscission, regulated by reactive oxygen species, sugar and phytohormones, signals cross-talk between fruitlet cortex and seed. It was also found that embryogenesis may block the consequent activation of the AZ^7^. Both shading and exogenous NAA in apples (*Malus domestica*) can result in apple abscission. Compared transcriptomics of both types of abscission indicated that shading-induced and NAA-induced abscission in apples results in reduced photosynthesis, alterations in carbohydrate transport, and hormone crosstalk^9^.

The increase of ROS-scavenging enzymes in tomato plants delayed the abscission of flowers and fruits, which indicated that there was a link between reactive oxygen species (ROS) and abscission^10^. In addition, the exposure of *Populus tremuloides* to ozone increased leaf abscission^11^. In ethylene-induced pedicel abscission of tobacco plants; the activity of peroxidase was increased^12^, indicating hydrogen peroxide has roles in leaf abscission^1^. An *in vitro* stress-induced leaf abscission system was established to identify the abscission signaling molecules. In this system, 1-mm-thick pulvinus strips, encompassing the AZ, were separated within 4 days of abscission at the AZ through cell wall degradation in an auxin depletion- and ethylene-dependent manner^1^. Using this system, Sakamoto *et al.*, (2008) demonstrated that hydrogen peroxide is involved in the abscission process in *Capsicum* plants. hydrogen peroxide is continuously produced at the *Capsicum* plants AZ throughout plant growth^1^. Pharmacological studies and gene expression analyses have strongly suggested that continuous hydrogen peroxide production at the AZ regulates cell wall-degrading enzymes gene expression^1^.

Cassava (*Manihot esculenta* Crantz) is a typical water-deficit tolerant plant that can tolerate long periods of water shortage^13^. The plant resists water-deficit stress by shedding older leaves and forming smaller new leaves, leading to adaptive water-deficit stress resistance^14^,^15^. The cassava plant has an easily observable AZ structure. This trait makes the cassava plant perfectly suited for the study of leaf abscission under stress. In this study we sought to understand the gene expression changes within this cellular structure that work to control leaf abscission. Microscopic observation indicated that cassava pulvinus AZ cell separation initiated at the later stages during water-deficit stress. A high throughput microarray containing 41,796 probe sets was constructed to identify and characterize the gene expression changes of AZ under water-deficit stress. The results of microarray indicated that the differential expression genes of AZ under water-deficit stress mainly participate in the pathways of response to oxidative stress, response to hydrogen peroxide, hydrogen peroxide catabolism, hydrogen peroxide biosynthesis, and oxidation reduction. Regulatory genes in reactive oxygen species related pathways involved in leaf abscission were identified. Potential reactive oxygen species changes in AZ indicated by the microarray were confirmed with microscopic observation, GC-MS analyses, and molecular research methods. Transgenic cassava plants, over-expressing cassava *SOD/CAT1* ROS-scavenging proteins, decreased the levels of hydrogen peroxide and ethylene in AZ under water-deficit stress, retarded the cell separation of the pulvinus AZ under water-deficit stress both *in vitro* and at the plant level. All the results indicated that ROS play important regulatory roles in the process of cassava leaf abscission under water-deficit stress. A preliminary model is presented to illustrate the process of cassava leaf abscission under water-deficit stress.

## Results

### The pulvinus AZ cell separation initiated at the later stages during water-deficit stress in cassava

**Figure 1:**
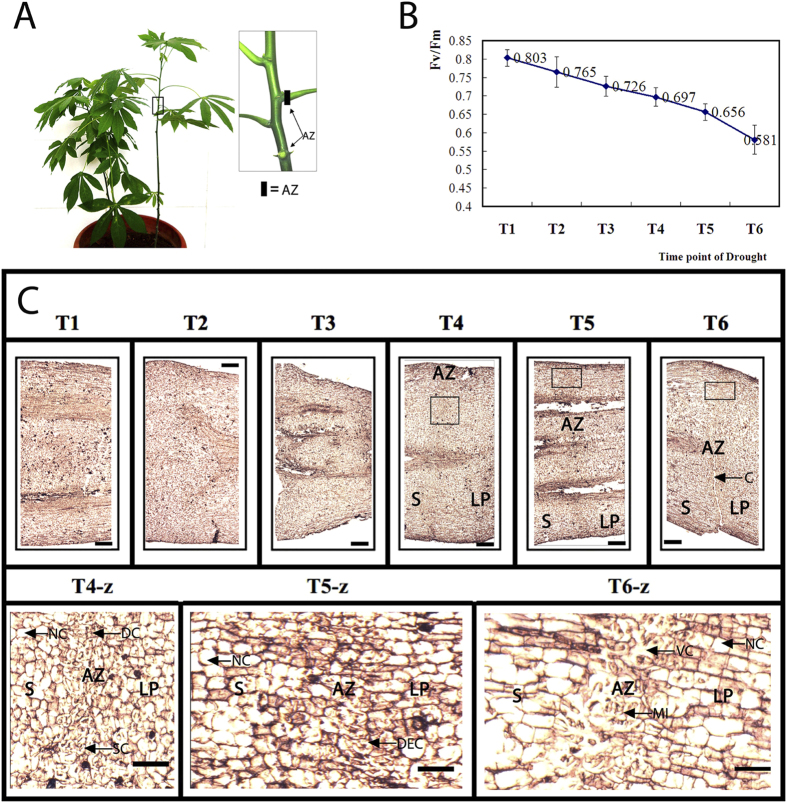
The pulvinus AZ cell separation appeared at the later stages under water-deficit stress in cassava. (**A**) Cassava plants and the pulvinus AZ. Schematic presentation of the cassava pulvinus AZ showed in the rectangles on the right side. AZ tissues selected as samples; (**B**) Fv/Fm was measured over 17 days, from Day 0, which was still well-watered. Six time points were selected during water-deficit stress. Three leaves in the middle of the cassava plants were sampled, with 3 cassava plants in a culture pot, and 5 pots for 1 treatment. The whole experiment was repeated 3 times. Each value represents the mean ± S.E. of 45 plants; (**C**) The process of the pulvinus abscission zone cell separation observed by microscope. The AZs were selected from leaves used for detection of Fv/Fm values and prepared in paraffin before section preparation and microscopic detection. T4-z, T5-z and T6-z represent, respectively, the rectangles shown in T4, T5 and T6. S: Stem, AZ: Abscission zone, LP: leaf petiole. SC: smaller cell, DC: dense cell, DEC: deformation cell, VC: vacuolar cell, MI: membrane invagination cell, C: crack, NC: normal cell. Bars in photographs T1 to T6 represent 1000 μm, Bars in photographs T4-z to T6-z represent 100 μm.

To create gene expression profiles for time course of cassava leaf abscission, physiological and anatomical characteristics at the pulvinus AZ in the cassava plant (Fig. 1A) were determined to define the AZ. T1, T2, T3, T4, T5 and T6 six time points were determined under water-deficit stress over 17 days according to the cassava leaf Chlorophyll fluorescence (Fv/Fm) values 0.803 ± 0.083, 0.765 ± 0.037, 0.726 ± 0.026, 0.697 ± 0.024, 0.656 ± 0.021 and 0.581 ± 0.041 respectively (Fig. 1B). AZ cell separation with microscopic observation under water-deficit stress was monitored over the 6 time points according to the Fv/Fm values (Fig. 1C). The AZ cell of the pulvinus in the first three time points (T1, T2 and T3) almost had normal shape and no abnormal cell were detected (Fig. 1C). However, the cell in the pulvinus AZ of the later three time points (T4, T5, and T6) under water-deficit stress became smaller, dense and serious deformation (Fig. 1C). The deformation, cytoplasmically smaller and dense cell almost arranged in a line throughout the transverse section of the pulvinus (Fig. 1C). Moreover, a crack in the AZ of the pulvinus was observed in the time point T6 (Fig. 1C). From the results of microscopy, no AZ specific cytoplasmically small and dense cells were found in the first three time point, but the stress induced cytoplasmically small and dense cells at the region where abscission will occur and crack at the generated AZ was observed indicated that the pulvinus AZ cell separation happened under water-deficit stress in cassava.

### Cassava leaf pulvinus AZ transcriptome evaluated by standard markers of cell-wall hydrolytic genes for abscission

**Figure 2:**
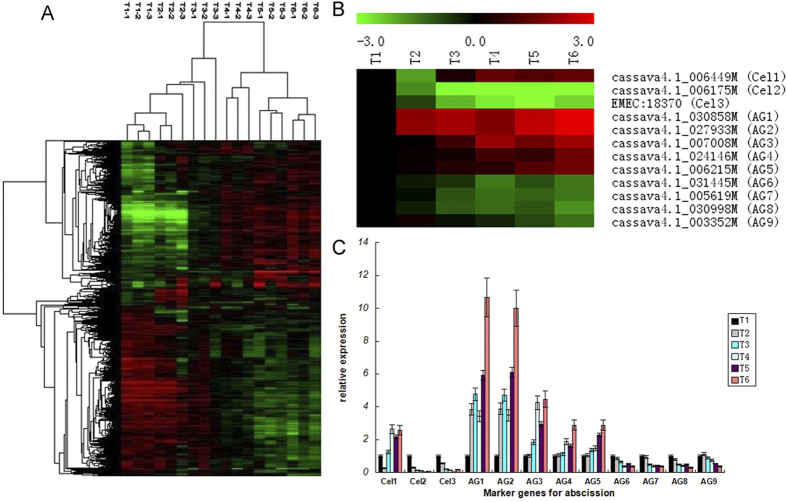
Time series cassava leaf pulvinus AZ transcriptome over six time points during leaf abscission. Hierarchical clustering analyses of differentially expressed genes. (**A**) The 10001 differentially expressed genes were clustered using hierarchical clustering. The signals are shown in a red-green color scale, where red represents higher expression and green represents lower expression. The numbers represent the time point during water-deficit stress and each sample was repeated three times; (**B**) The microarray expression ratios (*P* < 0.001) for marker genes involved in cell-wall hydrolysis during abscission, *cellulase* (*Cel*) or *polygalacturonase* (*AG*); (C) QRT-PCR analysis performed the expression patterns of *cellulase* (*Cel*) and *polygalacturonase* (*AG*). Relative expression levels were determined after normalizing all data to that of T1, which was set to 1.0. Error bars represent SD for three independent experiments.

Microarrays (NimbleGen) contained 41,796 probe sets, representing the 34,151 transcripts from the JGI database (http://www.phytozome.net/cassava.php) and the 7645 transcripts from Genbank database. Six time points of AZ samples defined with Fv/Fm values were analyzed by the cassava microarray with T1 time point as a control. The reliability of the microarray was evaluated by three internal controls housekeeping genes (*Histone 3*, *GAPC2*, and *Actin 7*). The expression signal intensity of the three housekeeping genes were consistent in the samples at different time points (Supplemental Fig. S1), which confirmed the reliability of these microarrays. The differentially expressed genes between tests (water-deficit stress, T2 to T6) and control (well-watered, T1) were analyzed with two class unpaired method in the Significant Analysis of Microarray software (SAM, version 3.02), false discovery rate (FDR) (<5%), and Wilcoxon Rank-Sum test (P < 0.01)^16^. The Hierarchical clustering of the differentially expressed genes indicated a clear separation of the samples from different time points and clustering of the 3 biological replicates of each time point, suggesting that the whole experiment from sample collection to data extraction was reproducible and reliable (Fig. 2A). The Hierarchical clustering indicated transcript expression patterns in time points T1, T2, and T3 had high similarity, and as did those in T4, T5, and T6. Moreover, the transcript expression patterns of the first three time points are completely opposite to the later three time points (Fig. 2A). The results indicated that the first three time points were growth states of the cassava AZs and that the last three time points were abscission states. The results of gene expression profliles in AZ indicated by Hierarchical clustering were consistent with the results of microscopic observation of AZ cells at six time point.

To evaluate the genes in regulating abscission presented in our transcriptome, we firstly analyzed those standard maker genes that involved in cell-wall hydrolysis during abscission, such as *cellulase* and *polygalacturonase*^17^,^18^. 3 *cellulase* and 9 *polygalacturonaseare* were detected in our transcriptome (Fig. 2B). Quantitative real-time PCR (qRT-PCR) confirmed the results of microarray on the expression patterns of *cellulase* and *polygalacturonase* genes, 1 *cellulase* and 5 *polygalacturonase* genes were greatly induced in the process of leaf abscission, while 2 *cellulase* and 4 *polygalacturonase* genes were down-regulated (Fig. 2C). The standard marker genes of abscission widely presented in the transcriptome confirmed the reliablity and creditability of the trascriptome profiling data.

### Four main expression patterns detected with significant expression changes in six time point during abscission with transcriptome profiling analyses

**Figure 3:**
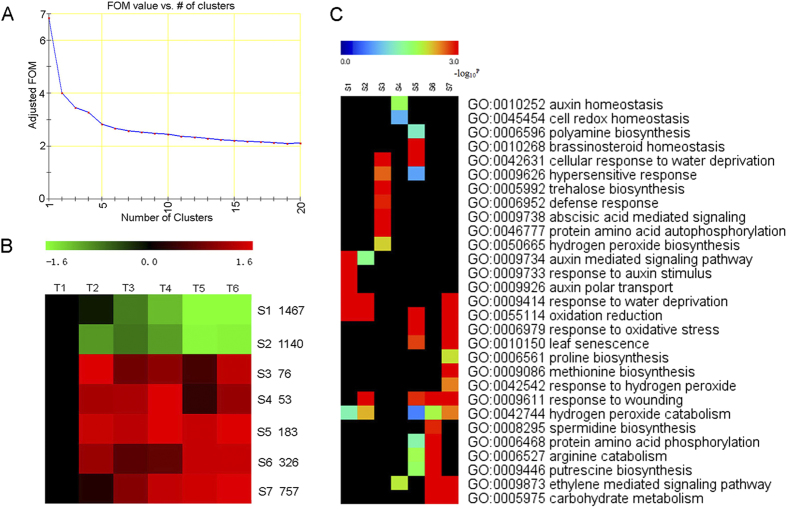
Dynamic progression of leaf abscission zone transcriptome under water-deficit stress. (**A**) SOTA clustering was performed with the MEV program (http://www.tm4.org/mev). The number of clusters (7 clusters) was defined by the FOM (Figures of Merit) application; (**B**) SOTA clustering showing the expression profile of the cassava leaf abscission zone transcriptome during water-deficit stress. SOTA clusters showed 4002 transcripts that have FDR-corrected P values <0.01 and fold change >2 or <0.5 in at least one of the time points under water-deficit stress. Seven clusters were defined by the FOM (Figures of Merit) application; (**C**) Functional classification using gene ontology annotation among the seven clusters (S1–S7). The genes in each cluster were then classified into the GO biological process categories. The enrichment of GO biological process related to each profile pattern after calculating, normalizing, transforming to log scale and clustering. The color reflects the enrichment levels of all genes found in each of these GO biological processes.

Self-organising Tree Algorithm (SOTA) clustering showed that FDR-corrected *P* values of <0.01 and significant expression changes occurred for at least one time point for each of the 4002 transcripts (Supplemental Data S1) under water-deficit stress. Seven (S1–S7) SOTA clusters (Supplemental Data S2) were divided into four main expression patterns (Fig. 3A,B), with T1 as control. The first type of expression pattern, including clusters S1 and S2, is down-regulated throughout the experimental period while compared to the control. The second type of expression pattern, including clusters S3, is up-regulated and with a higher expression level in the early experimental period (T2). The third type of expression pattern, including clusters S4 and S5, detected with higher expression level in the middle experimental period (T3, T4). The fourth type of expression pattern, including clusters S6 and S7, is up-regulated throughout the experimental period but with higher expression level at T5 and T6. (Fig. 3B).

### GO annotation indicated intense oxidation reduction-related genes involved in proline, polyamine, hydrogen peroxide and ethylene pathways accumulated in cassava pulvinus AZ under water-deficit stress

The differentially expressed genes of seven SOTA clusters were analyzed using GO (Gene Ontology) annotation (Supplemental Data S2 and S3) to identify the biological processes in which they function (Fig. 3B,C and Supplemental Data S3). Genes in the auxin mediated signaling pathway, oxidation reduction, auxin polar transport were greatly enriched in the clusters S1 and S2, representing genes down-regulated throughout the experimental period, indicating that the levels of auxin were changed in the AZ throughout the water-deficit stress period (Fig. 3B,C and Supplemental Data S3). Genes involved in response to water deprivation, defense response and hydrogen peroxide biosynthesis (cluster S3) showed peak expression at T2 under water-deficit stress, indicting that hydrogen peroxide and defense mechanism may be involved in the response to the early stages of water-deficit stress (Fig. 3B,C and Supplemental Data S3). Genes that showed peak expression at T3 and T4 are required for cell redox homeostasis, polyamine biosynthesis, auxin homeostasis, oxidation reduction and hydrogen peroxide catabolism (cluster S4, S5), indicating that intense oxidation reactions and hormone signaling occur in the AZ and that the levels of ROS and hormone changed at the middle stage of water-deficit stress (Fig. 3B,C and Supplemental Data S3). Genes involved in proline biosynthesis, polyamine (arginine, putrescine, spermidine) biosynthesis, hydrogen peroxide catabolism, ethylene mediated signaling pathway, oxidation reduction, response to wounding and carbohydrate metabolism (cluster S6 and cluster S7) reached their peak expression at T5 and T6. This indicated that the levels of polyamine, hydrogen peroxide, ethylene and carbohydrate varied in the AZ with the continuation of water-deficit stress (Fig. 3B,C and Supplemental Data S3).

### Proline-, polyamine-, hydrogen peroxide-, and ethylene-related biosynthesis and metabolism genes expressed with higher level in AZ than in non-separating tissues adjacent to the AZ

Since GO annotation revealed that many of the genes differentially expressed during leaf AZ cell separation under water-deficit stress were related to hydrogen peroxide, polyamine, proline, ethylene and oxidation reduction, the key genes involved in the biosynthesis of proline, polyamine, hydrogen peroxide, and ethylene were further analyzed using FDR-corrected P values < 0.001 as the significance criterion (Table 1)^19^. The results indicated that two genes involved in proline biosynthesis (*P5CS2-1* and *P5CS2-2*), two genes for polyamine biosynthesis (*ADC1* and *ADC2*), two genes involved in polyamine oxidative catabolism to produce hydrogen peroxide (*PAO1-1* and *PAO1-2*), and three genes for ethylene biosynthesis (two *ACO* genes and one *ACS* gene) were upregulated significantly in at least one of the time points during leaf abscission process under water-deficit stress.

We obtained complete cDNA sequences from plasmids containing genes encoding enzymes responsible for key steps in the biosynthesis of proline, polyamine, hydrogen peroxide and ethylene to compare to the GenBank annotations of similar genes from other plant species (Supplemental Table S1). The cassava P5CS2-1 and P5CS2-2 20,21 predicted amino acid sequences had 78% sequence identity with the *Arabidopsis* P5CS2 protein^22^. The two cassava genes *ADC1* and *ADC2*^23^,^24^ yield amino acid sequences 73% and 75% identical to the *Arabidopsis* ADC1 and ADC2 proteins, respectively. The two *PAO* genes^25^,^26^, *PAO1-1, PAO1-2*, yield amino acid sequences of 44% and 79% sequence identity with the *Arabidopsis* PAO proteins, respectively. There are two *ACO* genes and one *ACS* gene^27^,^28^ involved in the ethylene biosynthesis. The amino acid sequences of the three genes have 72%, 76% and 67% sequence identity with the *Arabidopsis* ACO and ACS proteins, respectively.

**Figure 4:**
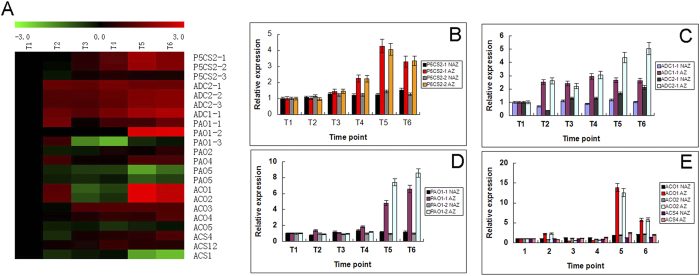
Detailed expression profiles of genes identified in microarray involved in biosynthesis of proline, polyamine, hydrogen peroxide, and ethylene in AZ and non-separating tissues adjacent to the AZ (Non-AZ). (**A**) shows the microarray expression ratios (*P* < 0.001) for key genes involved in biosynthesis of proline, polyamine, hydrogen peroxide, and ethylene; (**B**), (**C–E**) show QRT-PCR analysis the expression patterns of proline-, polyamine-, hydrogen peroxide-, and ethylene-related biosynthesis genes in AZ and non-separating tissues adjacent to the AZ (Non-AZ). Relative expression levels were determined after normalizing all data to that of T1, which was set to 1.0. Error bars represent SD for three independent experiments.

Quantitative real-time PCR (qRT-PCR) confirmed the results of microarray on the expression patterns of the important genes involved in the biosynthesis of proline, polyamine, hydrogen peroxide, and ethylene pathways (Fig. 4). To identify these genes expression pattern in both AZ cells and non-separating tissues adjacent to the AZ (Non-AZ), the comparative expression patterns of these genes between AZ cells and non-separating tissues adjacent to the AZ also confirmed by qRT-PCR. The expression levels of *P5CS2-1* and *P5CS2-2* both in AZ and Non-AZ were almost the same in T1, T2 and T3 time point, while detected with higher levels in AZ than Non-AZ in T4, T5 and T6 time point (Fig. 4B). The transcripts levels of *ADC1-1* and *ADC2-1* were with higher expression levels in AZ than Non-AZ under water-deficit stress, and reached peak values at later stages in AZ (Fig. 4C). The expression patterns of *PAO1-1* and *PAO1-2* genes, both involved in polyamine oxidative catabolism and hydrogen peroxide biosynthesis, detected with higher levels in AZ than Non-AZ, and reached the highest expression levels in AZ at later stage of water-deficit stress compared with at T1 (Fig. 4D). The relative expression levels of *ACO1*, *ACO2*, and *ACS4* in AZ and Non-AZ were almost the same at the early and middle stages under water-deficit stress, however, the expression patterns of three genes in AZ increased sharply at later stages under water-deficit stress while compared to that of Non-AZ (Fig. 4E).

### Microscopy and GC-MS suggest a role for hydrogen peroxide maybe produced by polyamine oxidization in leaf abscission

**Figure 5:**
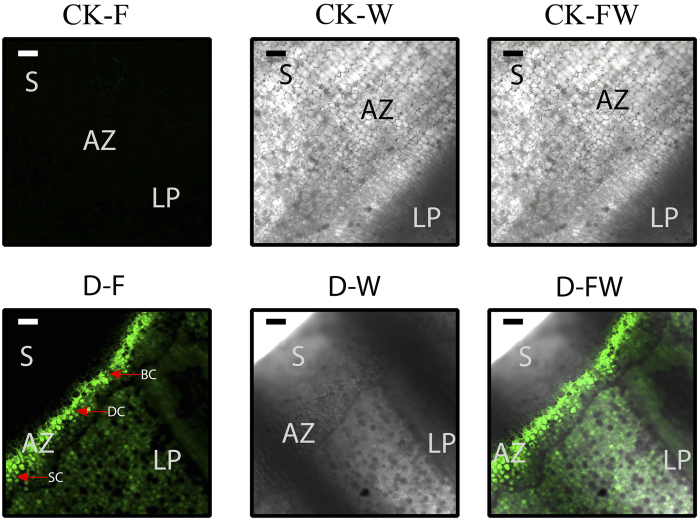
Hydrogen peroxide production during abscission under water-deficit stress as detected by dichlorofluorescein (DCF) fluorescence. The AZs observed with an Olympus FluoView™ FV1000 confocal microscope using a FITC filter. CK: AZ of normally watered cassava plants (T1) detected with DCF fluorescence (CK-F), bright-field (CK-W) and merged (CK-FW). (**D**) AZ at T5 under water-deficit stress cassava plants detected with DCF fluorescence (**D**–**F**), bright-field (D-W) and merged (D-FW). Scale bars: 250 μm. S: Stem, AZ: Abscission zone, LP: leaf petiole. BC: broken cell, DC: dense cell, SC: small cell.

Since the results of microarray indicated that many of the differential expression genes of AZ under water-deficit stress mainly participate in the pathways of response to hydrogen peroxide biosynthesis, hydrogen peroxide, hydrogen peroxide catabolism and oxidation reduction (Fig. 3C), which indicated that hydrogen peroxide maybe act an important role during AZ cell separation under water-deficit stress. To determine whether hydrogen peroxide participates in leaf abscission, microscopic examination of DCF fluorescent produced by hydrogen peroxide was carried out. Dichlorofluorescein diacetate (DCFH-DA) reacts with hydrogen peroxide in living cells to produce fluorescent DCF^1^. AZ samples were collected to correspond to time point T5 and at T1 as control. The excised AZs were treated with DCFH-DA solution for 1 h, then observed with an Olympus FluoView™ FV1000 confocal microscope. There were obviously cytoplasmically smaller cell, cytoplasmically dense cell, and cytoplasmically broken cell at the T5 sample under water-deficit stress, while the cell in CK sample had normal shape, no cytoplasmically smaller cell, cytoplasmically dense cell, and cytoplasmically deformation cell were detected (Fig. 5). Moreover, an obvious DCF signal was detected in the AZ at T5 sample treated with DCFH-DA solution. No fluorescent signals were observed in AZ at T1 treated with DCFH-DA (Fig. 5).

**Figure 6:**
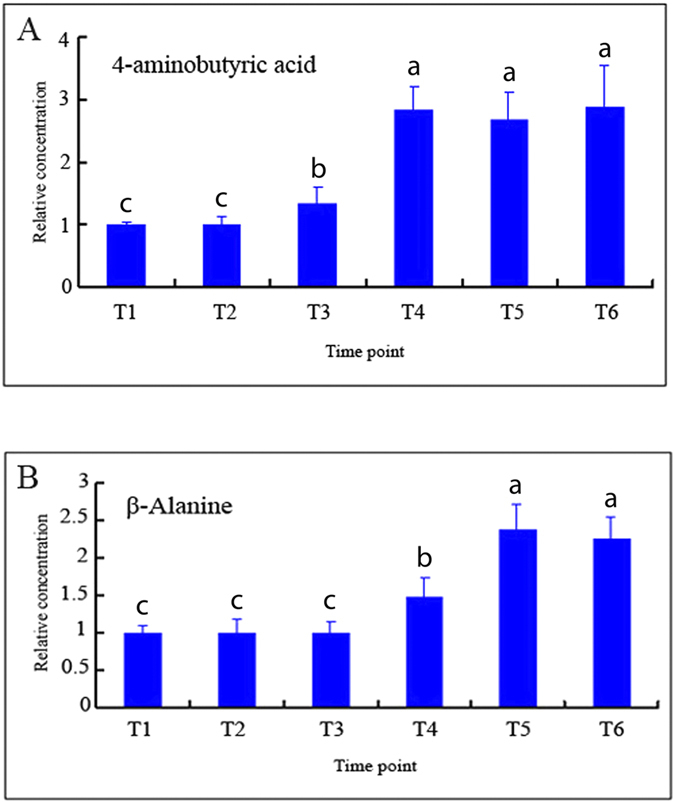
Polyamine oxidization catabolites 4-aminobutyric acid and β-alanine as side products of hydrogen peroxide production as detected by GC-MS in cassava abscission zone under water-deficit stress. AZs sample from water-deficit stress were taken according the Fv/Fm values, with T1 still under watered conditions. AZs in the middle of the cassava plants were sampled, with 3 cassava plants in a culture pot, and 5 pots for 1 treatment. The whole experiment was repeated 6 times. Each value represents the mean ± S.E. of 90 plants. Values labeled with different letters (a, b, and c) are significantly different by Duncan’s multiple comparison tests at P < 0.05. (**A**) 4-Aminobutyric Acid; (**B**) β-Alanine.

Polyamine oxidization by polyamine oxidase (PAO) produces both hydrogen peroxide and 4-aminobutyric acid^29^,^30^. To some extent, the level of the 4-aminobutyric acid can correlate to the level of hydrogen peroxide that produced by polyamine. The levels of 4-aminobutyric acid in AZ under water-deficit stress were detected by GC-MS (Fig. 6A, Supplemental Fig. S2), the results indicated that the concentration of 4-aminobutyric acid were significantly different among the six time points during leaf abscission. The level of 4-aminobutyric acid increased from T3 to T6, the higher levels of 4-aminobutyric acid appeared at the time point T4, T5 and T6 (Fig. 6A). β-Alanine, another catabolite of polyamine oxidization by polyamine oxidase, can also be quantified by GC-MS. β-Alanine levels rose at the later three time points, with the highest expression level at T5 (Fig. 6B). The levels of both 4-aminobutyric acid and β-Alanine byproducts indicated that hydrogen peroxide in cassava AZ under water-deficit stress is one products of the polyamine oxidization by PAO.

### GC-MS indicated that polyamine maybe produced by proline degradation in leaf abscission

**Figure 7:**
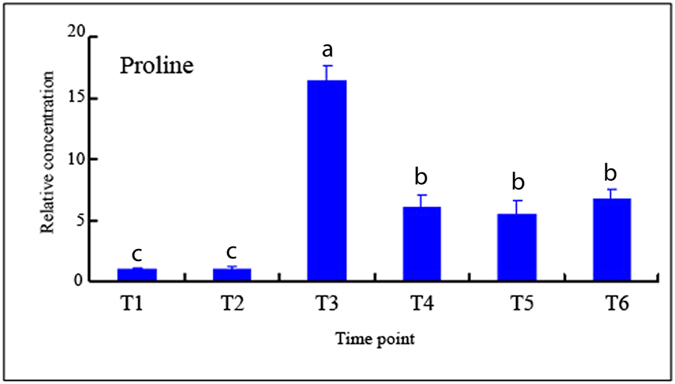
Proline content as detected by GC-MS in cassava abscission zone under water-deficit stress. AZs sample from water-deficit stress were taken according the Fv/Fm values, with T1 still under watered conditions. AZs in the middle of the cassava plants were sampled, with 3 cassava plants in a culture pot, and 5 pots for 1 treatment. The whole experiment was repeated 6 times. Each value represents the mean ± S.E. of 90 plants. Values labeled with different letters (a, b, and c) are significantly different by Duncan’s multiple comparison tests at P < 0.05.

Increase in proline is a common result when plants suffer stress^31^,^32^. Proline is one of the precursors of polyamine biosynthesis^32^,^33^. Regulation of the polyamine pathway by the proline was also reported^33^. To determine whether the proline biosynthesis genes that were upregulated in the microarray (Fig. 3C) that contribute to promote the proline level in the AZ under water-deficit stress. Proline content in AZ under water-deficit stress was measured with GC-MS (Fig. 7, Supplemental Fig. S2). Proline content in the AZ showed significant differences, it increases and achieves its highest level at T3 ((Fig. 7).

### Hydrogen peroxide significantly decreased in AZ of ROS scavengers *MeCu/ZnSOD* and *MeCAT1* transgenic cassava under water-deficit stress

**Figure 8:**
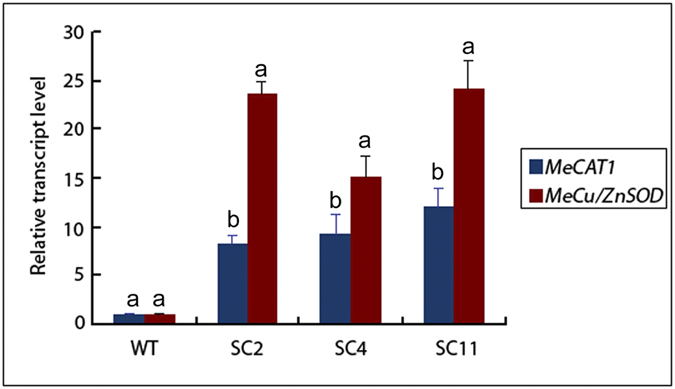
qRT-PCR analysis of *MeCu/ZnSOD* and *MeCAT1* expression levels both in wild-type (WT) and transgenic cassava lines. Total RNA was extracted from abscission zones. qRT-PCR data are shown relative to the wild type, using β-actin as an internal control. Data are presented as means ± SD of three independent RNA samples. SC 2: transgenic cassava plants that overexpress SOD/CAT1 proteins line 2; SC 4: transgenic cassava plants that overexpress SOD/CAT1 proteins line 4; SC 11: transgenic cassava plants that overexpress SOD/CAT1 proteins line 11.

If ROS are indeed involved in the abscission process as hypothesized, plants expressing ROS-scavenging genes should demonstrate repression of AZ cell separation. The eight single-copy transgenic cassava lines overexpressing the ROS scavengers *MeCu/ZnSOD* and *MeCAT1* produced by Xu *et al.*, (2013a) were used to test ROS regulation of AZ cell separation^34^. Quantitative reverse transcription (qRT)-PCR (Supplemental Table S2) showed that the expression levels of *MeCu/ZnSOD* and *MeCAT1* in the AZ of cassava line SC2, SC4, and SC11 were about 20- and 10- higher than WT cassava respectively (Fig. 8).

**Figure 9:**
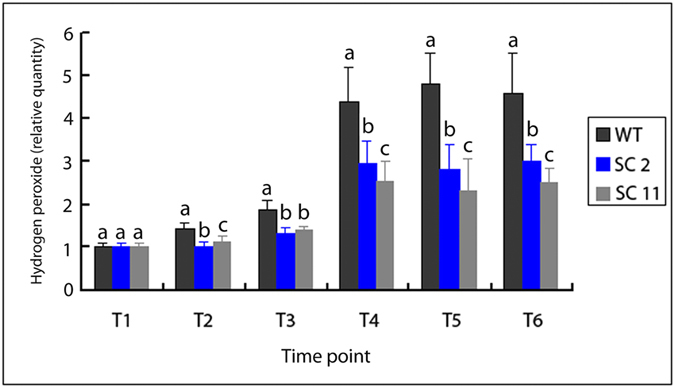
The levels of hydrogen peroxide in wild type and in transgenic cassava plants that overexpress SOD/CAT1 proteins. AZs were sampled in the middle of the cassava plants, with 3 cassava plants in a culture pot, and 5 pots for 1 treatment. The whole experiment was repeated 3 times. Each value represents the mean ± S.E. of 45 plants. Values labeled with different letters (a, b, and c) are significantly different by Duncan’s multiple comparison tests at P < 0.05. WT: wild type cassava plants, SC 2: transgenic cassava plants that overexpress SOD/CAT1 proteins line 2; SC 11: transgenic cassava plants that overexpress SOD/CAT1 proteins line 11.

To confirm whether the levels of hydrogen peroxide are different between in *MeCu/ZnSOD* and *MeCAT1* transgenic cassava plants and WT plants at the plant level, the measure of hydrogen peroxide contents in *MeCu/ZnSOD* and *MeCAT1* transgenic cassava plants and WT plants were carried out. The levels of hydrogen peroxide in AZ at six time points under water-deficit stress in *MeCu/ZnSOD* and *MeCAT1* transgenic cassava plants and WT plants were detected with DAB (3, 3′-diaminobenzidine) staining and colorimetric detection. The results of the DAB staining and colorimetric detection indicated that the levels of hydrogen peroxide significantly different in AZ among the six time points either in *MeCu/ZnSOD* and *MeCAT1* transgenic cassava plants or WT plants during water-deficit stress. The level of hydrogen peroxide increased from T3 to T6, the higher levels of hydrogen peroxide appeared at the time point T4, T5 and T6 in both type of transgenic plants and WT. However, the levels of hydrogen peroxide in *MeCu/ZnSOD* and *MeCAT1* transgenic cassava plants decreased significantly in time point T4, T5 and T6 while compared with WT (Fig. 9).

### AZ cell separation retarded in *MeCu/ZnSOD* and *MeCAT1* transgenic plants under hydrogen peroxide treatment

**Figure 10:**
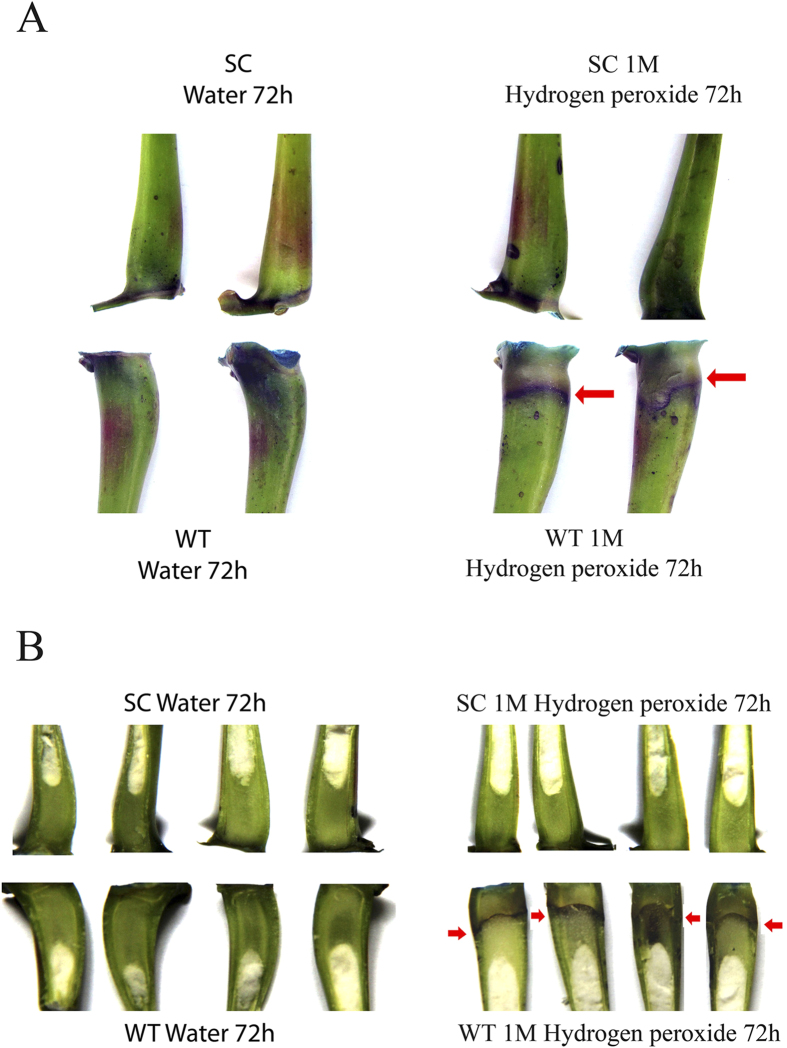
The pulvinus AZ cell separation retarded detected by Evans blue (EB) staining in *MeCu/ZnSOD* and *MeCAT1* transgenic plants under oxidative stress. (**A**) cassava abscission zones after 72 h-treatment with water or 1 M hydrogen peroxide followed by EB staining for wild type (WT, lower) and transgenic (WT, lower) plants. Arrows mark the position of the AZ that stained with EB staining; (**B**) Longitudinal sections of abscission zones after 72 h-treatment with water or 1 M hydrogen peroxide followed by EB staining for wild type (WT, lower) and transgenic (WT, lower) plants. Arrows mark the position of the AZ that stained with EB staining.

To confirm the transgenic plants have the function to affect AZ cell separation by scavenge hydrogen peroxide in cassava plant, either 1 M hydrogen peroxide or water as a control were used to treat the AZs of wild type and transgenic plants. After 72 h treatment, cell death in the AZ of wild type and transgenic cassava plants was detected using viability staining with Evans Blue (EB), a dye that is taken up specifically by dead cells^35^. In the AZs of wild type plants, no EB staining was detected after 72 h treatment with water (Fig. 10). However, there was obvious EB staining observed when the wild type was treated with 1 M hydrogen peroxide for 72 h, which indicated that the cell separation of abscission was initiated (Fig. 10). In the AZs of transgenic plants, treatment with water resulted in a similar lack of staining (Fig. 10). Interestingly, when the AZs of transgenic plants were treated with 1 M hydrogen peroxide, there was also no EB staining observed (Fig. 10). This suggested that the AZ cell separation was blocked in the transgenic plants.

### Microscopic observation of blocked AZ cell separation in transgenic cassava plants overexpressing Cu/ZnSOD/CAT1 ROS-scavenging proteins

**Figure 11:**
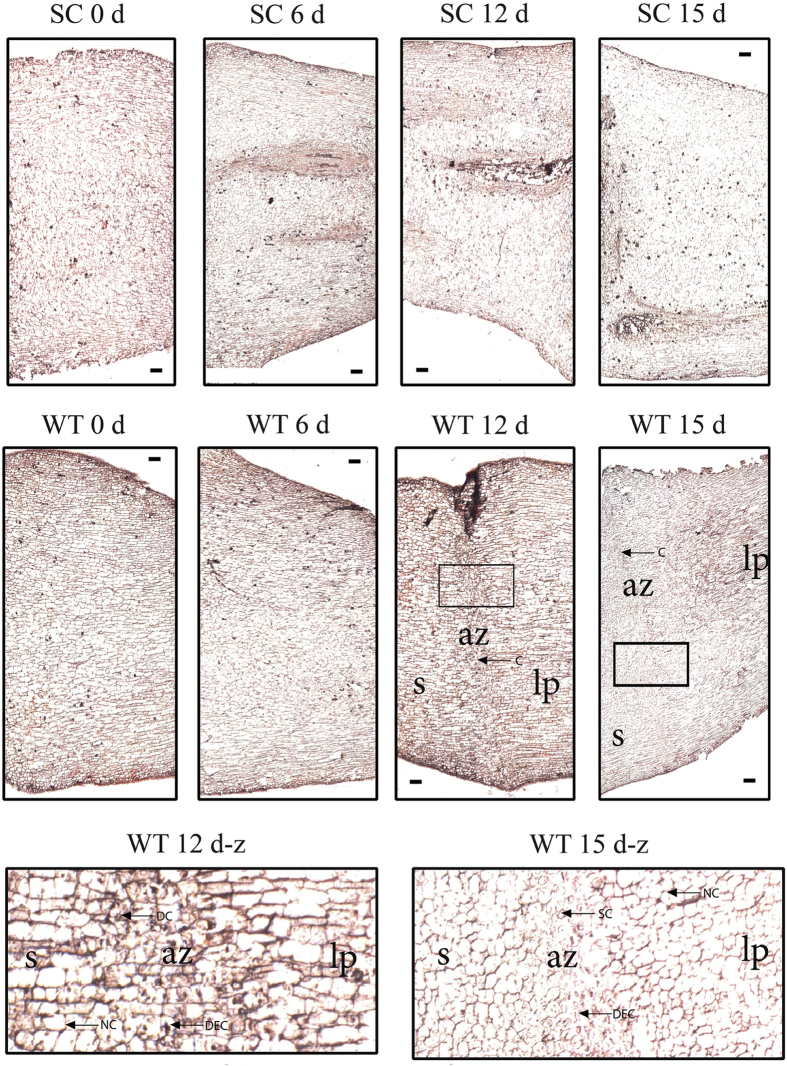
Progression of abscission zone cell separation in transgenic cassava plants. The abscission zones of wild type (WT) and transgenic lines over-expressing *SOD/CAT1* (SA) at four stages (water-deficit stress in 0 d, 6 d, 12 d and 15 d) were selected for paraffin section preparation and microscopic detection. WT 12 d-z and WT 15 d-z represent the boxed parts of panels WT 12 d and WT 15 d. S: Stem, AZ: Abscission zone, LP: leaf petiole. NC: normal cell, DC: dense cell, DEC: deformation cell, SC: smaller cell, C: crack. Bars in photographs represent 500 μm.

In transgenic cassava plants overexpressing SOD/CAT1 ROS-scavenging proteins shows enhanced resistance to water-deficit stress, and less leaves were dropped off in transgenic cassava plants under 30 days dehydration treatment while compared to WT cassava plants^36^. To understand whether AZ cell separation had different between transgenic plants and WT, paraffin sections were carried out to detect the cell separation in AZ of both transgenic plants and WT under water-deficit stress. Paraffin sectioning and microscopic observation of cells at the AZ were carried out after 0 d, 6 d, 12 d, and 15 d of water-deficit stress for transgenic and wild type plants. In the over expressing *SOD/CAT1* ROS-scavenging protein transgenic cassava plants, the cell in the AZ of the pulvinus have normal shape, no obvious deformation cell and no crack was detected in the four time points during water-deficit stress (Fig. 11), indicated that there were no cell separation in the AZ of the transgenic plants under 15 days water-deficit stress. In the wild type cassava plants, there was no AZ cell separation in either 0 d or 6 d under water-deficit stress (Fig. 11). However, many cytoplasmically dense cell, smaller cell and deformation cell were detected either in 12d or 15 d water-deficit stress (Fig. 11). Moreover, the cracks were obviously observed either in 12d or 15 d water-deficit stress (Fig. 11). Those results form wild type plants indicated that the cell separation in the AZ of wild type cassava plants happen in the later period of the water-deficit stress.

### Ethylene biosynthesis gene and ethylene levels decreased in AZ of *MeCu/ZnSOD* and *MeCAT1* transgenic plants

**Figure 12:**
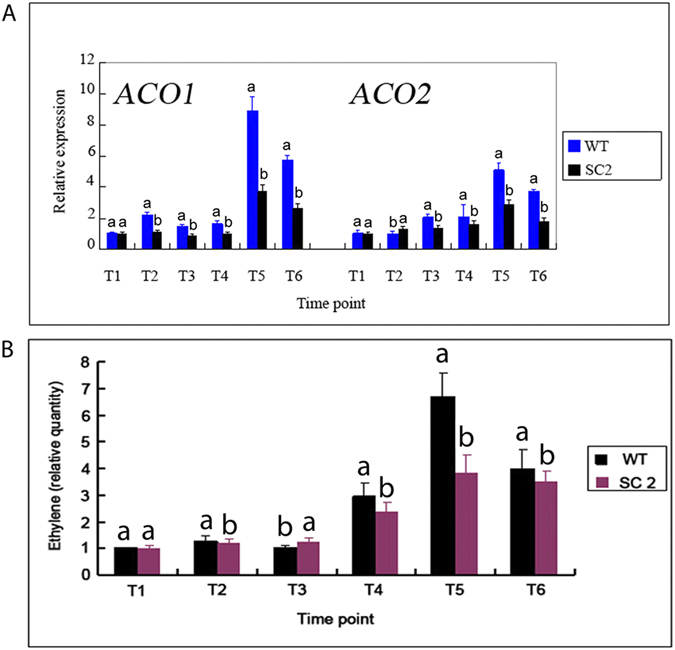
The expression patterns of ethylene biosynthesis-related genes and levels of ethylene in wild type and transgenic cassava plants that over express SOD/CAT1 proteins. (**A**) Expression patterns of ethylene biosynthesis *ACO* genes in WT and transgenic plants, RNA was extracted and subjected to qRT-PCR using primers specific to *ACO1* or *ACO2* over the course of water-deficit stress. RNA was extracted independently 3 times; (**B**) The levels of ethylene in WT and transgenic plants. Ethylene concentration over six time points under water-deficit stress as examined by GC analysis. AZs in the middle of the cassava plants were sampled, with 3 cassava plants in a culture pot, and 5 pots for 1 treatment. The whole experiment was repeated 3 times. Each value represents the mean ± S.E. of 45 plants. Values labeled with different letters (a, b, and c) are significantly different by Duncan’s multiple comparison tests at P < 0.05.

The expression levels of two *ACO* genes involved in ethylene biosynthesis were analyzed in the wild type and transgenic cassava plants. The qRT-PCR results indicted that the expression levels of both *ACO* genes were lower in transgenic plants compared to the wild type plants (Fig. 12A).

GC analysis was used to correlate up-regulation of *ACO* genes with ethylene concentration over the six time points during leaf abscission. The AZs were ground and used for ethylene concentration analysis. In WT plants, Higher levels of ethylene appeared at the later stages of leaf abscission, from T4 to T6, with the highest ethylene concentration at T5, of about 7 folds compared to the control T1 (Fig. 12B). In *MeCu/ZnSOD* and *MeCAT1* transgenic plants, the levels of ethylene increased gradually, and reach the peak at the T5 time point, about 4 folds compared to the control T1 (Fig. 12B). These results clearly indicated that ethylene contents decreased in transgenic plants compared to the WT plants.

## Discussion

In our study, transcriptomic, genetic, cellular, molecular, metabolomic, physiological and transgenic methods were used to study cassava AZ cell separation, with all results indicating that ROS pathway has an important regulatory role in cassava leaf abscission under water-deficit stress. Our result is consistent with previous report^1^ that ROS acts as an abscission signaling to promote cell separation in AZ under *in vitro* stressed cells. Several studies have shown that hydrogen peroxide, produced from polyamine degradation by polyamine oxidase gene (*PAO*), can induce hypersensitive cell death^37^,^38^. Spermidine oxidization into hydrogen peroxide through *PAO* can also be induced by the salinity in the apoplast of tobacco transgenic plants overexpressing apoplastic polyamine oxidase, the accumulated hydrogen peroxide results in the induction of either tolerance responses or programmed call death (PCD) depend on the levels of intracellular PAO proteins^39^. Tisi et al., (2011) also proved that over-expressed *PAO* or increased spermidine supply enhanced *in vivo* hydrogen peroxide production in plant tissues. hydrogen peroxide derived from polyamine catabolism behaves as a signal for secondary wall deposition and for induction of PCD^40^. Besides hydrogen peroxide, other ROS can act as signals modulating plant PCD^41^. Bar-Dror et al., (2011) discovered that ROS resulted in PCD was proved to regulate abscission in tomato^35^. All these results indicate that hydrogen peroxide produced from polyamine oxidation by *PAO* genes involved in the process of PCD in plant cells. Our results also supported all these reports. The results of AZ cell separation with microscopy observation indicated that PCD happened in AZs of cassava plants under water-deficit stress (Figs 1 and 10). DCF detection of hydrogen peroxide confirmed that hydrogen peroxide exists in the AZ during the process of leaf abscission (Fig. 5). Moreover, in overexpressing *SOD/CAT1* ROS-scavenging proteins transgenic cassava plants, hydrogen peroxide decreased significantly in AZ of transgenic cassava plants while compared to WT under water-deficit stress, indicating that hydrogen peroxide involved in regulating leaf abscission under water-deficit stress. In addition, the results of both microarray and QPCR showed up-regulation of the genes for polyamine conversion to hydrogen peroxide (Fig. 4D) and further confirmed the higher expression levels of *PAOs* in AZ compared with Non-AZ. The results of GC-MS confirmed that 4-aminobutyric acid (Fig. 6A) and β-Alanine (Fig. 6B) involved in the process of leaf abscission under water-deficit stress. Both compounds are byproducts in the process of polyamine oxidation into hydrogen peroxide^25^,^30^. 4-aminobutyric acid and β-Alanine detected in AZ under water-deficit stress suggested that polyamine oxidation into hydrogen peroxide occurred in the process of cassava leaf abscission.

**Figure 13:**
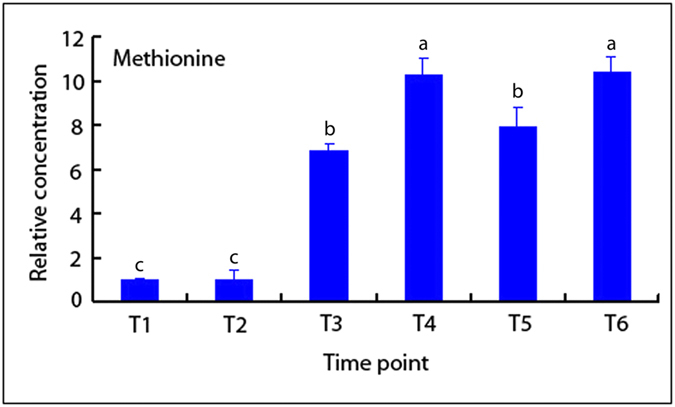
Methionine content as detected by GC-MS in cassava abscission zone under water-deficit stress. AZs sample from water-deficit stress were taken according the Fv/Fm values, with T1 still under watered conditions. AZs in the middle of the cassava plants were sampled, with 3 cassava plants in a culture pot, and 5 pots for 1 treatment. The whole experiment was repeated 6 times. Each value represents the mean ± S.E. of 90 plants. Values labeled with different letters (a, b, and c) are significantly different by Duncan’s multiple comparison tests at P < 0.05.

Several reports show that polyamines not only regulate hydrogen peroxide biosynthesis, but also modulate ethylene pathway^42^. Another link is through S-adenosylmethionine (SAM), which acts both as a precursor for ethylene production and also as a substrate in the biosynthesis of polyamines^43^,^44^. Methionine (Met) is the precursor of SAM. We detected a large Met pool by GC-MS in the process of leaf abscission (Fig. 13, Supplemental Fig. S2). The existence of a large Met pool indicated that the cellular pool of S-adenosylmethionine is probably large enough to satisfy the demand for polyamine and ethylene production, reducing competition between the two pathways^45^.

Proline is one of the precursors of polyamine biosynthesis^33^. Regulation of the polyamine pathway by the proline was also reported^33^. Proline content detected with GC-MS indicated that proline contenet increases and achieves its highest level at T3 ((Fig. 7). However, the results of both the microarray and quantitative PCR showed that proline biosynthesis *P5S2* genes were up-regulated under water-deficit stress, and higher expression levels emerged at the later stages of abscission in AZ (Fig. 4B). The expression patterns of *P5S2* genes were inconsistent with the results of GC-MS on proline content indicated that proline degradation into polyamine may be occurring at the later stages of water-deficit stress in AZ.

In overexpressing *SOD/CAT1* ROS-scavenging proteins transgenic cassava plants, either the level of ethylene biosynthesis genes or ethylene concentration in AZ was down-regulated (Fig. 12A,B), which indicated ROS-scavenging proteins can decrease ethylene biosynthesis genes expression and ethylene produce. In fact, there are feedback/feedforward interactions between ROS and ethylene occur in plant 46. Ethylene accumulation can be induced by hydrogen peroxide^46^. Ethylene also can be induced by promote the level of hydrogen peroxide through reducing peroxisomal catalase activity in tobacco plants as an earliest response of high light irradiance^46^. Furthermore, ethylene production can be increased by exogenous application of hydrogen peroxide in pine needles in a concentration-dependent manner^46^. In addition, Ozone, which is known to form ROS in apoplast, induces accumulation of ethylene in tobacco plants^46^. Ozone-induced leaf damage is preceded by a rapid increase in ACC synthase activity, ACC content and ethylene produce, which related to ROS accumulation^47^,^48^ In addition, the ethylene receptors genes were induced differently by Ozone in tomato^48^. ROS can promote ethylene produce, inversely; ethylene also can regulate the level of ROS. The research of co-localization predicts that high concentrations of both ethylene and ROS occur in the same cells in a temporally coordinated manner^48^. For example, in tomato, both ethylene biosynthesis and hydrogen peroxide accumulation were confined to the parenchyma cells of vascular tissue^48^. Ethylene has a potentiating role in oxidative cell death by controlling O_2−_ accumulation^47^,^48^. In addition, the lack of ethylene synthesis and AOX induction caused increased ROS production^47^,^48^.

**Figure 14:**
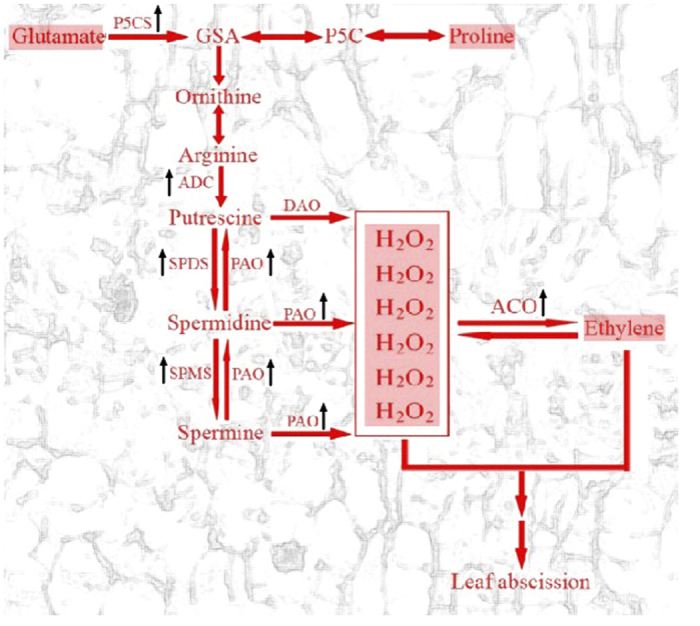
Schematic illustration of the intrinsic relationship between proline, polyamine, ROS, and ethylene that regulates leaf abscission zone development. Arrows show those genes that upregulated in the AZs by microarray and Q-PCR; shadowy parts show those compounds that identified with high levels in the AZs under water-deficit stress by the GC-MS and GC.

In conclusion, we used transcriptome, anatomical, physiological, biochemical and molecular methods confirmed that oxidative stress may enhance abscission related gene expression, cause morphological and physiological transition, and finally shed the stressed leaves. This also confirmed by transgenic cassava over-expressing redox enzymes MeCu/ZnSOD and MeCAT1. Cassava AZ cell separation in response to water-deficit stress indicated that the molecular controls are held by proline, polyamines, ROS, and ethylene (Fig. 14). Under water-deficit stress, the accumulated proline and polyamines contributed to the promotion of ROS in abscission zones, all these factors induced ethylene biogenesis, which promotes abscission related gene expression and resulted in leaf abscission (Fig. 14).

## Materials and Methods

### Plant material and treatments

Cassava cultivar SC5 was grown as described previously^49^. Three-month-old cassava plants with a uniform growth status were chosen for water-deficit stress treatments. Water-deficit stress tolerance of the cassava plants was assessed in terms of the maximum photochemical efficiency of chlorophyll fluorescence parameter Fv/Fm in the middle of the cassava leaves^50^. Fv/Fm were detected at 0 days under water-deficit stress and continued until cassava leaf abscission (about 17 days after water-deficit stress). Six time points were selected to collect AZ samples according to Fv/Fm values. About 1–2 millimetres of each pulvinus (Fig.1A), including AZ, were cut for sample collection. Because of the stable ethylene concentration in the middle of the plants, pulvinuss were sampled from the middle parts of the cassava plants^51^. AZs about 1–2 mm in size were frozen in liquid nitrogen for RNA extraction. The RNA was purified using the Plant RNA Reagent (Invitrogen), resulting in a pure and high-quality RNA preparation, based on spectroscopic and gel electrophoresis analyses. For *MeCu/ZnSOD* and *MeCAT1* transgenic cassava plants and WT plants, the transgenic cassava lines overexpressing the ROS scavengers MeCu/ZnSOD and MeCAT1 produced by Xu *et al.*, (2013a) were used to test ROS regulation of AZ cell separation^34^. Each pot contained 3 clones when we planted cassava plants and 5 pots were prepared for 1 treatment and repeated 3 times, 1 treatment with 3 times repetition regarded as one biological replicate, three biological replicates were used for ROS detection analysis.

### Paraffin section and microscopic observation

Paraffin sectioning was used to detect the histology of cassava leaf AZ as Zhou *et al.*, (2011) described previously^52^. Briefly, sections (about 10 μm thick) of the AZs were cut with a microtome (Leica RM2245), and stained with 1% (w/v) safranin O (Amresco) and 1% (w/v) fast green FCF (Merck), examined with a fluorescence microscope (Zeiss AXIO Imager A1), and photographed^52^.

### The cassava whole genome microarray: design, hybridization, and data analysis

Cassava time series whole genome microarray experimental design based on the principle of the “loop design” was performed as described by Breeze *et al.* (2011)^53^. Total RNA was extracted with Plant RNA Reagent (Invitrogen): the purity and quality was assessed by formaldehyde agarose gel electrophoresis and the quantity was determined spectrophotometrically.

Two public databases were used for cassava microarray construction: the great majority of the ESTs originated from JGI database (http://www.phytozome.net/cassava.php) and the minority based on sequences from NCBI with E <1e-5. Custom-designed 60-mer Nimblegen DNA microarrays were synthesized by maskless *in situ* photolithographic synthesis^54^. The fluorescent dye (Cy3-dCTP)-labeled cassava cDNA was produced as previously described using CapitalBio cRNA Amplification and Labeling Kit (CapitalBio). After completion of double-stranded cDNA (dsDNA) synthesis, the dsDNA products were purified using a PCR NucleoSpin Extract II Kit (MN). The resulting cRNA was labeled according to Nimblegen recommendations^54^. The procedures of Array hybridization, washing, scanning and data analysis were performed at CapitalBio Corporation (Beijing, China) according to the NimbleGen’s Expression user’s guide. The expression data of probes were normalized using quantile normalization and expression data of genes were generated using the Robust Multichip Average (RMA) algorithm^55^.

### Time course analysis

For comparison analysis, differential gene expression between samples (T2 to T6) and control (T1) was identified by Significant Analysis of Microarray software (SAM, version 3.02)^16^,^53^. The differentially expressed genes exceeding a threshold fold change >2.0 or <0.5, a Wilcoxon Rank-Sum test significance level at 0.01 (P < 0.01), and a threshold of false discovery rate (FDR) <1% in the SAM output were considered significant. The time-dependent differentially expressed genes were classified by SOTA clustering with the MeV 4.0 software^56^. The Hierarchical cluster analysis of the differentially expressed genes was clustered with Cluster 3.0 software.

### GO analysis

GO annotation analyses on gene clusters were performed using BiNGO as described by Maere *et al.* (2005)^57^. Significance GO categories were identified using a hypergeometric test with a significance threshold of 0.01 after a Benjamini and Hochberg FDR correction^58^. GO categories were classified by Hierarchical cluster with the MeV 4.0 software.

### Quantitative real-time PCR

RNA was extracted from cassava leaf AZ (about 1–2 mm long) and non-separating tissues adjacent to the AZ at the different time points according to the Fv/Fm values during water-deficit stress. The expression pattern analyses by QPCR were repeated three times in the six different samples that were used for the microarray analysis. Gene-specific primers were designed using IDT Primerquest tools (http://www.idtdna.com/Primerquest/Home/Index) as listed in Supplemental Table S2 online. The SYBR Green PCR kit (Applied Biosystems) was used to perform QPCR^19^. The QPCR procedures were as follows: 10 min of denaturation at 95 °C, followed by 40 cycles of amplification with 15 sec of denaturation at 94 °C, 30 sec of annealing according to the melting temperatures provided in Supplemental Table S2 online, 35 sec of extension at 72 °C, and the fluorescence data collection at 72 °C. After a final extension at 72 °C for 10 min, the specificity of the amplified product was evaluated by melting curve^19^. For *MeCu/ZnSOD* and *MeCAT1* transgenic plants analyses, the molecular analyses of transgenic plants were carried out as described by Xu *et al.*, (2013a). In detail, the expression levels of *MeCu/ZnSOD* and *MeCAT1* transgenes were determined by real-time qRT-PCR. The primers used in transgenic identification are listed in Supplemental Table S2.

### Cassava AZ intracellular hydrogen peroxide detection

Cassava leaf AZs about 2 mm long were cut from both water-deficit stress-treated and well-watered cassava plants. The AZs of cassava plants at T5 time point under water-deficit stress were selected as sample. The cassava AZ intracellular hydrogen peroxide was detected with DCFH-DA as previously described^1^ with minor modification. The AZs were incubated in 50 μM DCFH-DA, dissolved with DMSO, 1 h. All the AZs of samples and controls were rinsed with PBS buffer, then were observed with a Olympus FluoView™ FV1000 confocal microscope and FITC filter for detection the DCF fluorescence in the AZ cells.

### GC-MS

Cassava leaf AZ (about 1–2 mm thick) used for GC-MS analyses were collected at six time points according to the Fv/Fm values during water-deficit stress. AZs in the middle of the cassava plants were sampled, with 3 cassava plants in a culture pot, and 5 pots for 1 treatment. The whole experiment was repeated 6 times. GC-MS analyses, including metabolite extraction, derivatization, GC-MS analysis, and data processing, were performed as described previously^59^. Metabolites were identified in comparison to database entries of authentic standards^59^.

### Hydrogen peroxide measurement in AZ of wild type plants and *MeCu/ZnSOD*, *MeCAT1* transgenic plants

Colorimetric analyses were carried out as described by Xu *et al.*, (2013a) with DAB staining^34^. In detail, one gram of AZ tissues of transgenic plants and WT plants was homogenized in an ice bath with 10 mL of 0.1% TCA. Then, the homogenate centrifuged at 10,000 g for 20 min for acquiring the supernatant. One milliliter of the supernatant was added to 1 mL of 10 mM potassium phosphate buffer (pH 7.0) and 2 mL of 1 M potassium iodide. The absorbance of the supernatant was read at 390 nm. The quantity of hydrogen peroxide was determined from the standard curve.

### Oxidative stress experiments *in Vitro* for AZ both in wild type plants and *MeCu/ZnSOD*, *MeCAT1* transgenic plants

Two wild type and two transgenic plants were planted in the same pot, after 3 months, the pulvini, including AZs in the middle of the plants, were selected for sample collection. The pulvini with leaves were cut from the stems. The base of the pulvini was immersed into a water or a 1 M hydrogen peroxide (in water) solution. After 72 h, the cell viability of the AZ was evaluated by Evens blue (EB) staining^35^. Briefly, the base of the pulvini was stained with 0.2% EB solution for 5 min. After staining, the base of the pulvini were washed with frequent water changes for at least 30 min, and photographed.

### Endogenous ethylene measurements in wild type plants and *MeCu/ZnSOD*, *MeCAT1* transgenic plants

Endogenous ethylene measurements were carried out as described by Shi *et al.* (2006)^19^. Cassava leaf AZ tissue samples (5 g tissues about 50 AZs) at six time points were collected, the samples were ground in liquid nitrogen and sown in 1-liter airtight glass jars. Air samples (100 mL) from each flask were removed and injected into the column of the gas chromatograph for ethylene measurements. A gas chromatograph (GC-14C; Shimadzu) equipped with a flame-ionization detector with a 30-m HP-PLOT column (Agilent Technologies) was used to measure the amount of ethylene produced^19^. Standards of 0.1, 1, 10, and 50 ppm ethylene were used to verify the retention time and to quantify the amount of ethylene produced^19^. The concentration of ethylene of T1 sample acts as a control.

## Additional Information

**How to cite this article**: Liao, W. *et al.* Reactive oxygen species regulate leaf pulvinus abscission zone cell separation in response to water-deficit stress in cassava. *Sci. Rep.* **6**, 21542; doi: 10.1038/srep21542 (2016).

## Supplementary Material

Supplemental Data S1

Supplemental Data S2

Supplemental Data S3

Supplementary Information

## Figures and Tables

**Table 1:** FDR-corrected P values for cDNAs encoding key enzymes in various proline, polyamine, hydrogen peroxide, and ethylene pathways.

Array ID	Enzyme	FDR-Corrected P Value^a^					P < 0.05	P < 0.01
		T2/T1	T3/T1	T4/T1	T5/T1	T6/T1		
Proline biosynthesis								
002371M	P5CS2	0.6852	0. 3555	0.0064	0.0000	0.0012	Y	Y
002374M	P5CS2	0.6353	0.4193	0. 0265	0.0025	0.0047	Y	Y
002381M	P5CS2	0.5577	0.6379	0.1239	0.2897	0.2934	Y	N
Polyamine biosynthesis								
002501M	ADC2	0.0073	0.0069	0.0004	0.0027	0.0013	Y	Y
002558M	ADC2	0.1210	0.1213	0.0124	0.0054	0.0016	Y	Y
2428(501)	ADC2	0.0444	0.0365	0.0031	0.0007	0.0001	Y	Y
9782(558	ADC1	0.0276	0.0302	0.0004	0.0000	0.0000	Y	Y
Hydrogen Peroxide biosynthesis								
006541M	PAO1	0.0135	0.6932	0.0097	0.0122	0.0122	Y	Y
023906M	PAO1	0.0000	0.0000	0.0000	0.0000	0.0000	Y	Y
032640M	PAO1	0.3677	0.0195	0.0010	0.2977	0.2977	Y	N
006326M	PAO2	0.4545	0.5404	0.4878	0.4661	0.4661	N	N
005130M	PAO4	0.1122	0.6686	0.5816	0.0397	0.0397	N	N
004753M	PAO5	0.3517	0.1687	0.0583	0.0005	0.0005	Y	Y
034376M	PAO5	0.3845	0.2255	0.4588	0.0148	0.0148	Y	N
Ethylene biosynthesis								
012494M	ACO1	0.4545	0.0322	0.1118	0.0000	0.0004	Y	Y
012045M	ACO2	0.6852	0.3894	0.0164	0.0636	0.0013	Y	Y
012052M	ACO	0.4473	0.5589	0.1238	0.2447	0.4275	N	N
012282M	ACO	0.6353	0.0000	0.0004	0.0004	0.0002	Y	Y
14555	ACO1	0.4545	0.0522	0.2434	0.0000	0.0004	Y	N
011508	ACS4	0.1443	0.5986	0.0555	0.0248	0.0004	Y	N
005191	ACS12	0.6602	0.5262	0.0186	0.0812	0.1076	N	N
006665	ACS1	0.2587	0.4829	0.5339	0.0000	0.0000	Y	Y

^a^FDR-Corrected P Values < 1e-6 were shown as 0.
