# Supplementary material for: Reactive oxygen species regulate leaf pulvinus abscission zone cell separation in response to water-deficit stress in cassava
Source: Sci Rep. 2016 Feb 22;6:21542. doi: 10.1038/srep21542 (PMC4761936; doi:10.1038/srep21542)
Supplement: Supplementary Information [file srep21542-s4.pdf]

**Supplemental Information**

**Title: Reactive oxygen species regulate leaf pulvinus abscission zone cell separation in response to water-deficit stress in cassava**

**Authors:**

**Wenbin Liao**

Institute of Tropical Bioscience and Biotechnology, Chinese Academy of Tropical Agricultural Sciences, Haikou 571101, China

**Peng Zhang**

National Key Laboratory of Plant Molecular Genetics and National Center for Plant Gene Research (Shanghai), Institute of Plant Physiology and Ecology, Shanghai Institutes for Biological Sciences, Chinese Academy of Sciences, Shanghai 200032, China

**Gan Wang**

Institute of Tropical Bioscience and Biotechnology, Chinese Academy of Tropical Agricultural Sciences, Haikou 571101, China

**Yayun Li**

Institute of Tropical Bioscience and Biotechnology, Chinese Academy of Tropical Agricultural Sciences, Haikou 571101, China

**Jia Xu**

National Key Laboratory of Plant Molecular Genetics and National Center for Plant Gene Research (Shanghai), Institute of Plant Physiology and Ecology, Shanghai Institutes for Biological Sciences, Chinese Academy of Sciences, Shanghai 200032, China

**Jianbo Sun**

Institute of Tropical Bioscience and Biotechnology, Chinese Academy of Tropical Agricultural Sciences, Haikou 571101, China

**Ming Peng**

Institute of Tropical Bioscience and Biotechnology, Chinese Academy of Tropical Agricultural Sciences, Haikou 571101, China

Email: mingpengcatas@gmail.com

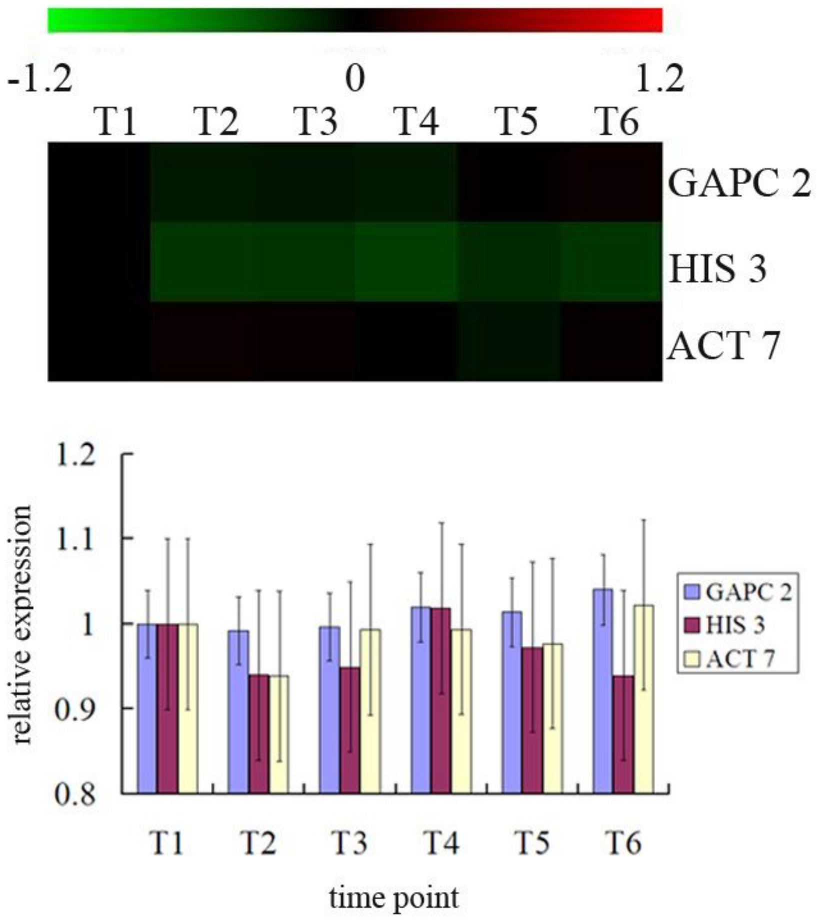


**Supplemental Figure S1 | Quantitative PCR analysis confirmed the reliability and accuracy of microarray by three housekeeping genes (*Histone 3*, *GAPC2*, and *Actin 7*).**

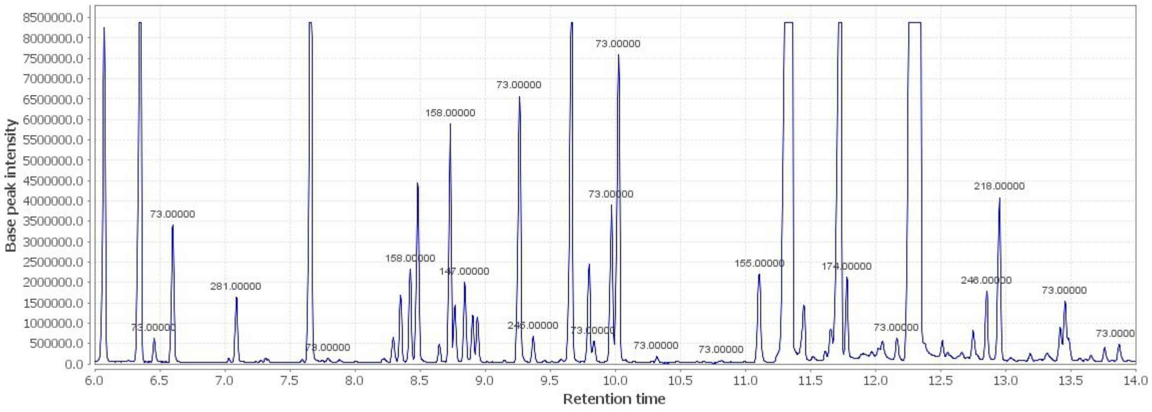


Representative total ion current chromatogram (TIC) of time point T1 of drought condition

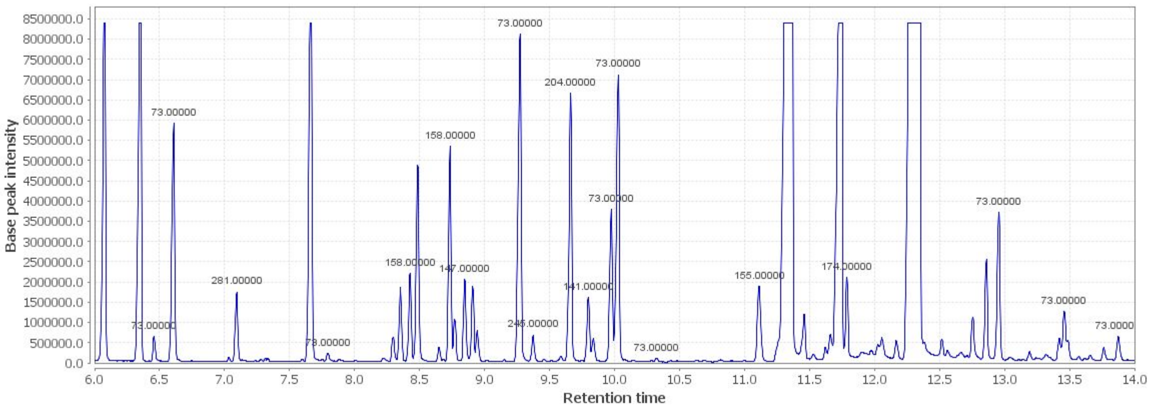


Representative total ion current chromatogram (TIC) of time point T2 of drought condition

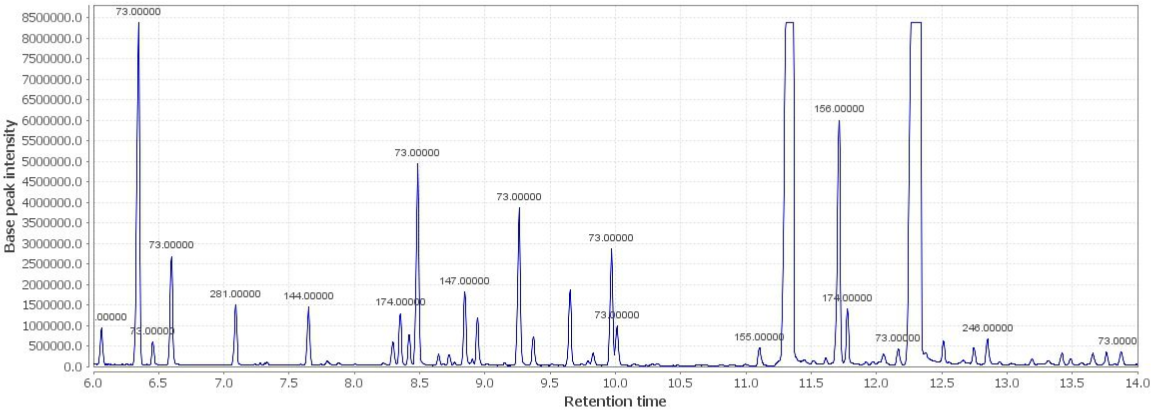


Representative total ion current chromatogram (TIC) of time point T3 of drought condition

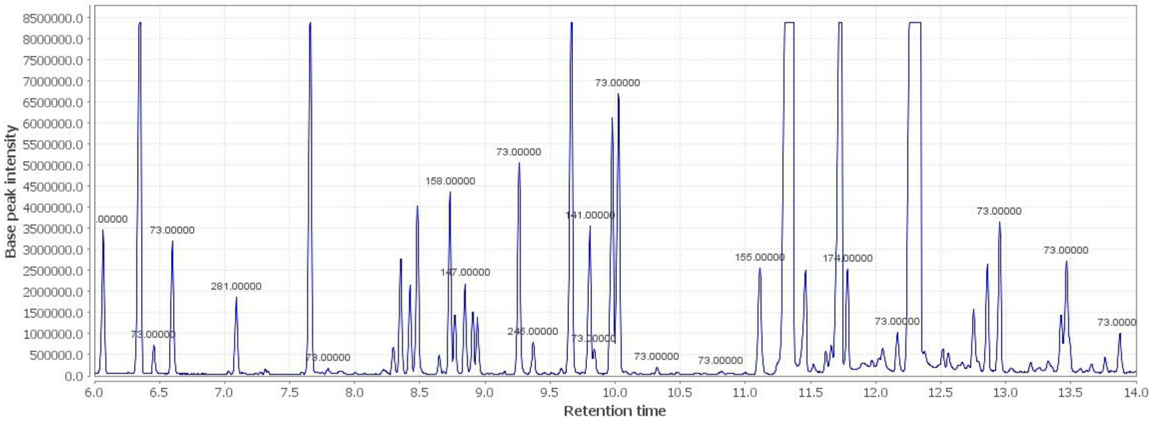


Representative total ion current chromatogram (TIC) of time point T4 of drought condition

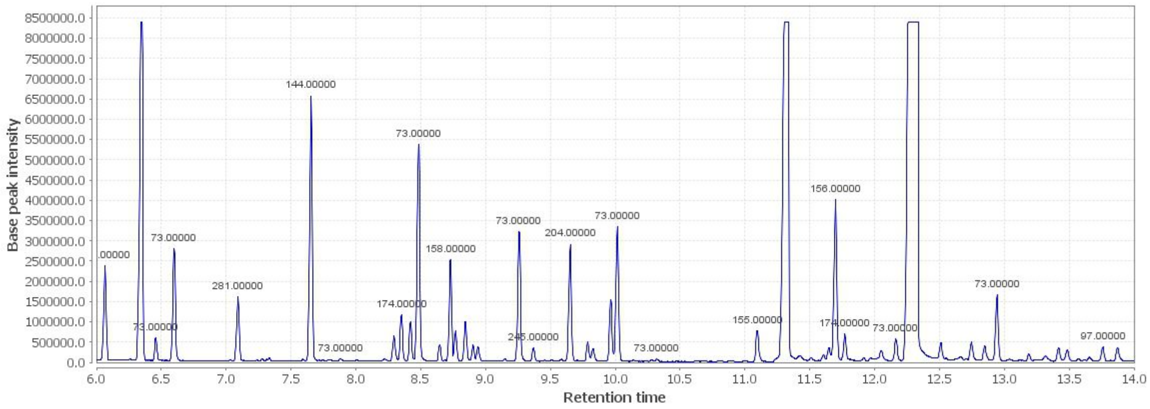


Representative total ion current chromatogram (TIC) of time point T5 of drought condition

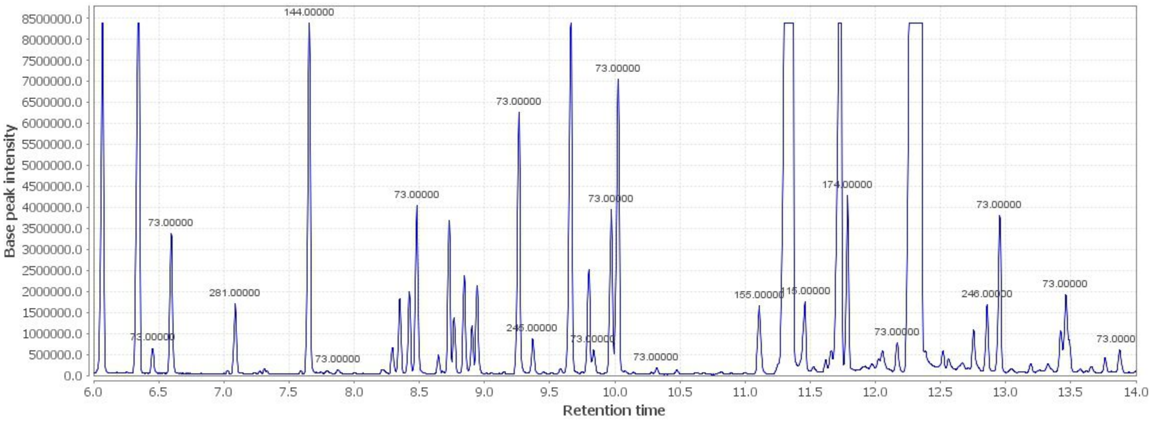


Representative total ion current chromatogram (TIC) of time point T6 of drought condition

**Supplemental Figure S2 | Representative total ion current chromatogram (TIC) of six time point under drought.**

**(Alanine 6.08， Aspartic acid 9.98， Succinic acid 11.37，**

**L-Methionine 11.65， L-Proline 11.72，4-aminobutyric acid 11.78，**

**Succinic acid 12.75， Glutamic acid 12.86， Glucose. 16.10)**

**Supplemental Table 1 | cDNAs Encoding Key Enzymes in Various Plant Proline, Polyamine, Hydrogen Peroxide, Ethylene Biosynthetic Pathways**

| Array ID | Enzyme | ORF length (bp)[a] | Full (F) or Partial (P) ORFs | Best hit in NCBI[b] | Amino Acid identity (%) | Conserved Domain[c] | E-value |
|---|---|---|---|---|---|---|---|
| | | | Proline biosynthesis | | | | |
| 002371M | P5CS2 | 2220 | F | XP_002524230 | 89 | [d]cd04256; cd07079 | 4.65e-123 1.09e-177 |
| 002374M | P5CS2 | 2220 | F | XP_002524230 | 89 | cd04256; cd07079 | 4.65e-123 1.09e-177 |
| 002381M | P5CS2 | 2217 | F | XP_002524230 | 89 | cd04256; cd07079 | 1.48e-120 0e+00 |
| | | | Polyamine biosynthesis | | | | |
| 002501M | ADC2 | 2175 | F | XP_002306141 | 83 | cd06830 | 8.86e-133 |
| 9782 | ADC1 | 2175 | F | XP_002306141 | 81 | cd06830 | 1.44e-134 |
| | | | Hydrogen Peroxide biosynthesis | | | | |
| 006541M | PAO1 | 1455 | F | XP_002511416 | 81 | pfam13450 | 1.85e-11 |
| 023906M | PAO1 | 1482 | F | XP_002517783 | 92 | pfam13450 | 8.76e-05 |
| 004753M | PAO5 | 1680 | F | XP_002514111 | 78 | pfam13450 | 2.33e-09 |
| | | | Ethylene biosynthesis | | | | |
| 012494M | ACO1 | 921 | F | AAX84675 | 99 | pfam14226 pfam03171 | 1.57e-17 9.12e-30 |
| 012045M | ACO2 | 957 | F | ABK58140 | 93 | pfam14226 pfam03171 | 4.45e-22 9.33e-37 |
| 011508M | ACS4 | 1002 | F | XP_002509645 | 85 | cd00609 | 2.15e-54 |

**[a]Length of the putative ORF of the cassava gene.**

**[b]The closest homolog exhibiting the lowest BLASTX E-value to the cassava**

**enzyme.**

**[c]The putative ORF of each cDNA was translated into amino acid sequence,**

**and conserved domains were searched using the BLASTP program.**

**cd04256:AAK_P5CS_ProBA, Glutamate-5-kinase (G5K) domain.**

**[d]cd07079: ALDH_F18-19_ProA-GPR, Gamma-glutamyl phosphate reductase (GPR), aldehyde dehydrogenase families 18 and 19.**

**cd06830: PLPDE_III_ADC, Type III Pyridoxal 5-phosphate (PLP)-Dependent Enzyme Arginine Decarboxylase.**

**pfam13450: NAD_binding_8, NAD(P)-binding Rossmann-like domain.**

**pfam14226: DIOX_N, non-haem dioxygenase in morphine synthesis N-termina.**

**pfam03171: 2OG-FeII_Oxy, 2OG-Fe(II) oxygenase superfamily.**

**cd00609: AAT_like, Aspartate aminotransferase family.**

## Supplemental Table S2 | Primers Used for QRT- PCR Analysis

| Genes | ID | Primer Designation | Sequences |
|---|---|---|---|
| *Cel1* | cassava4.1_006449M | Cel1-5’ | 5’-GCCAAAAGACAGGTGGACTACATC |
| | | Cel1-3’ | 3’-TTACTTCACCCTACTTTGGGTGGC |
| *Cel2* | cassava4.1_006175m | Cel2-5’ | 5’-GCTAAACGTCAGGTGGACTACATTC |
| | | Cel2-3’ | 3’-GGTGACCAATTCGCGCACCACTTAC |
| *Cel3* | EMEC:18370 | Cel3-5’ | 5’-TGGGAGAAAATCCAATGAGGATGT |
| | | Cel3-3’ | 3’-ATTCGCGCACCACTTACAATATGA |
| *AG1* | cassava4.1_030858M | AG1-5’ | 5’-GAGAATTAAGACTTGGCCCGCATTG |
| | | AG1-3’ | 3’-GCTGCTGCACGTAATTTGAACGGC |
| *AG2* | cassava4.1_027933M | AG2-5’ | 5’-CCTATGCAATCGGAAGAAGCCATCA |
| | | AG2-3’ | 3’-CAATGTGCAACCGAGAAATTTTCTC |
| *AG3* | cassava4.1_007008M | AG3-5’ | 5’-TGCAGGGATTTCGAGTGATGTGACC |
| | | AG3-3’ | 3’-GCCTCACATATTGTGGATTAAGTTC |
| *AG4* | cassava4.1_024146M | AG4-5’ | 5’-CCTCAACTATCAGGCAAACAAGTTC |
| | | AG4-3’ | 3’-CAACAGTTCTCTCAGGCATAATATC |
| *AG5* | cassava4.1_006215M | AG5-5’ | 5’-GCAGCTAAGGCCAAGAAGATACCA |
| | | AG5-3’ | 3’-GGTAGAAGAACAAGTGTTTGCTCG |
| *AG6* | cassava4.1_031445M | AG6-5’ | 5’-CTGCCAAAACCAGACATCTGCTGTA |
| | | AG6-3’ | 3’-CTACATTCCACTTGCCCAATTTAG |
| *AG7* | cassava4.1_005619M | AG7-5’ | 5’-TAGACGTAAGCCTAACCACCATAG |
| | | AG7-3’ | 3’-CCGACCACACAAGTCAATACACC |
| *AG8* | cassava4.1_030998M | AG8-5’ | 5’-TTCCATGTGCAAACCAGACTTCAG |
| | | AG8-3’ | 3’-CATTATTCTGGGTAGAGTGGGTCC |
| *AG9* | cassava4.1_003352M | AG9-5’ | 5’-GTCACTTAGATACTTCGTCTTGGAG |
| | | AG9-3’ | 3’-CTCTGAAGATCGAGCTACCGGCTA |
| *P5CS2-1* | cassava4.1_002371M | P5CS2-1-5’ | 5’-AGGTGCAGGTCGGAATAAGTACA |
| | | P5CS2-1-3’ | 5’-CAGTGAATGCCAACGTCTCCAC |
| *P5CS2-2* | cassava4.1_002374M | P5CS2-2-5’ | 5’-TAGGTGCAGAGGTCGGAATAAGT |
| | | P5CS2-2-3’ | 5’-CCTCTCAGGATCCATCTTGTTGT |
| *ADC2-1* | cassava4.1_002501M | ADC2-1-5’ | 5’-GTAGTGATGTCCTCCGGGTTAT |
| | | ADC2-1-3’ | 5’-CGTTGCCATCGTCACTGTCTTC |
| *ADC2-2* | cassava4.1_002558M | ADC2-2-5’ | 5’-GATGACTACGCCTTTGGAAGATG |
| | | ADC2-2-3’ | 5’-CAATATCAACTCCAAGTCCAGGTA |
| *PAO1-1* | cassava4.1_006541M | PAO1-1-5’ | 5’-GGCATGTCAGGAATTTCAGCAGCA |
| | | PAO1-1-3’ | 5’-AAGGGATTGGCTACAGTTCCTCCA |
| *PAO1-2* | cassava4.1_023906M | PAO1-2-5’ | 5’-CTCAGGTATAGAAACGAGTAAATC |
| | | PAO1-2-3’ | 5’-CGATCCTGTCAAAGCTAGTAAGG |
| *ACO1* | cassava4.1_012494M | ACO1-5’ | 5’-ACAGGAATTTTGCAAATGGATGG |
| | | ACO1-3’ | 5’-TTCTCCAGACCAAGGTTCTCAC |
| *ACO2* | EMEC:14555 | ACO2-5’ | 5’-TGAGAGTCTACTTCCATTCTTGTC |
| | | ACO2-3’ | 5’-CATCAATACGGAGACCCAACT |
| *ACS4* | cassava4.1_011508M | ACS-4-5’ | 5’-GGGTGTCCTCCACTTCAAATTC |

| | | | |
|---|---|---|---|
| | | ACS-4-3’ | 5’-CGGCTTCTCTAAGGCCACTAAC |
| *ACT7* | cassava4.1_009780M | ACT7-5’ | 5’-CACCACCAGAGAGGAAGTATAG |
| | | ACT7-3’ | 5’-ACATCTGCTGGAAGGTGCTGAGG |
| *GAPC2* | cassava4_1_012218M | GAPC2-5’ | 5’-CTGCTATCAAGGAGGAATCTGAGG |
| | | GAPC2-3’ | 5’-CACGGGTGCTGTAGCCCCACTC |
| *HIS3* | cassava4.1_018853M | HIS3-5’ | 5’-CAGTGGCTCTTCGTGAGATTCGT |
| | | HIS3-3’ | 5’-ATCCGTCTTGAAGTCTTGTGCAAT |
| *APX2* | cassava4.1_013461M | APX2-5’ | 5’-GTCGTCTCCCTAATGCTACTAAAG |
| | | APX2-3’ | 5’-CCACCAGAAAGGACAACAATATC |
| *SOD* | cassava4.1_018289M | SOD-5’ | 5’-ATGCTGGTGATCTGGGAAATGT |
| | | SOD-3’ | 5’-GATATTCCTCTTTCTGGTCCGC |
